# Waste Study on Flexible Food and Non-Food Packaging: Detailed Analysis of the Plastic Composition of European Polyethylene-Containing Waste Streams

**DOI:** 10.3390/ma17133202

**Published:** 2024-06-30

**Authors:** Nelly Freitag, Johannes Schneider, Virginie Decottignies, Tanja Fell, Esra Kucukpinar, Martin Schlummer

**Affiliations:** 1 Fraunhofer Institute for Process Engineering and Packaging IVV, 85354 Freising, Germany; tanja.fell@ivv.fraunhofer.de (T.F.); esra.kucukpinar@ivv.fraunhofer.de (E.K.); martin.schlummer@ivv.fraunhofer.de (M.S.); 2 SUEZ S.A., 78230 Le Pecq, France; virginie.decottignies@suez.com

**Keywords:** selective dissolution, flexible packaging, sorting, recycling, polymers, packaging waste, composition analysis

## Abstract

Despite extensive sorting, packaging waste often contains a mixture of different materials that make high-quality recycling difficult, especially in the case of flexible packaging. This is partly due to the widespread use of multi-layer laminates and packaging consisting of different inseparably combined materials. To improve the post-consumer recyclate quality and develop optimised recycling processes, it is important to generate a comprehensive understanding of the composition of the sorted packaging waste streams. Therefore, in this study, polyolefin sorting fractions for flexible packaging waste from three European countries are analysed in detail. By selective extraction of the different plastics, their mass fractions in the waste streams are determined. This shows that the PE-rich sorting fractions for flexible packaging are made up of 85–90% of PE, but also contain a certain proportion of foreign materials. A detailed analysis of the layer structures of various types of packaging also provides information on the prevalence of multi-layer packaging and the polymer and non-polymer materials used therein. This shows that particularly in food packaging, with 63–84% of multi-layer and 50–70% of multi-material packaging, a high proportion of foreign materials is used and introduced into the sorting fractions. These insights provide a valuable contribution to the development of recyclable packaging, potential sorting streams and recycling processes, especially with regard to the challenges of the closed-loop recycling of food packaging.

## 1. Introduction

The need for packaging keeps increasing, indicating a shift towards more flexible packaging [1]. Plastics is a popular material solution with a 33% share of the worldwide consumer packaging market due to low oil and gas prices [2,3]. The main polymers used in flexible packaging are low-density (LDPE) and linear low-density polyethylene (LLDPE). PE alone does not fulfil all requirements needed in the large product range of packaging, which is why it is usually combined with other polymers in multi-layer structures, e.g., to reduce food losses and protect highly sensitive products [4,5]. However, out of the 3.2 Mio tons of plastics used for packaging in 2021 only in Germany, 3.19 Mio tons of waste were generated, of which only 55% were sent to recycling. The amount of recycled plastics that is produced from this and actually replaces virgin plastic is even significantly lower [6,7].

To potentially unify the current patchwork rug of recycling infrastructure, definitions, guidelines, and restrictions within Europe, the Packaging and Packaging Waste Directive (PPWD since 1994, turning into PPWR soon) introduces specific measures like deposit return schemes or incentives and also specifies material recycling and recyclate reuse targets for 2030 [8]. It is currently under review to be turned into a Regulation (PPWR) that will bring new binding rules for all European Countries and the European plastic packaging market. The Extended Producer Responsibility (EPR) is, for example, one measure to survey the plastic packaging put on the market and to ensure a takeback or recycling, because the packaging producers or brand owners pay for an adequate management of the waste arising from their products [9]. Currently, the EPR is managed on a national level, which leads to differences between the countries in Europe, e.g., in terms of waste definition, level of fees and data reporting [10]. The collection, sorting and recycling rates of post-consumer plastic packaging varies strongly between countries depending on the established collection schemes or the ban of landfill. On average, around 46% of post-consumer packaging waste in Europe was collected for recycling in 2020, but only 8.5% of new packaging products contain post-consumer recycled plastic [7]. The sorting follows a similar base scheme in all countries involving (1) bag opening; (2) separation of light goods (films) through wind sifting; (3) near-infrared (NIR) sorting. The number of sorting steps and sorting streams differs. In some facilities, NIR sorting is also used to add small flexible PE packaging that was collected in the heavy fraction during wind sifting back to a sorting fraction [11]. While the sorting residues are sent for energy recovery in France and Belgium, in Germany, the remaining flexible packaging waste is sorted further into additional mixed waste fractions, which has a positive effect on the recycling rate and the availability of secondary raw material [7].

To incorporate recycled material in packaging material, the value stream must comply with the strict safety regulation EC1616 (2022), especially when it comes to contact with sensitive products (EC 10/2011). Accordingly, it is necessary to assure a food contact compliance of the original input material and a “suitable recycling technology” to reuse the recyclate in contact-sensitive applications [12]. Flexible packaging often used for food applications is sorted to a less extent than rigid plastic packaging (e.g., made of high-density PE (HDPE) or PP). The combination of different polymers in the so-called multi-layer films is currently impossible to recycle into a secondary raw material of sufficient quality for similar packaging applications using the thermomechanical recycling processes established on the market. Instead, the material mix is either incinerated for energy recovery or, if sorted, used in applications with lower performance requirements [13,14,15].

As a foundation for improved sorting infrastructure and suitable recycling technologies, it is therefore necessary to gain a more general and comprehensive understanding of the composition of the current sorting fractions (with regard to flexible packaging) to unravel unused potentials [13,16]. Several studies have partially investigated the polymeric composition of waste streams. Roosen et al. analysed the composition of selected packaging samples of the most frequently sold products of Belgian food retailers using analytical methods (FT-IR, differential scanning calorimetry (DSC), polarised optical microscopy (POM)). This revealed the high diversity of different polymers and other materials used in food packaging, which are often inseparably combined as multi-material packaging [17]. CEFLEX started a study in 2020 with the aim of understanding the plastic packaging streams in Europe better. Their findings show that different amounts of flexible packaging end up in recyclable waste streams depending on the collection infrastructure and that between 40% and 60% of flexible packaging can be considered recyclable, e.g., ≥50% was found to be “mono-films” in France and Germany [18]. A dedicated differentiation between food and non-food packaging has not been performed.

This paper, therefore, focuses on the flexible packaging found in sorted PE-rich fractions and investigates large homogenised samples to build a solid base for future developments of the packaging design but also of sorting and/or recycling technologies. For the analysis, flexible packaging is initially categorised according to its use (food/non-food). The study then uses selective dissolution to separate the different polymers in flexible packaging waste. Selective solvents dissolve target plastics from the packaging surface and promote their diffusion through the outer polymer layers in the case they are insoluble, which leads to a breakdown of the interaction between the polymer layers. Due to the delamination of the multi-layer structure, the solvents can interact with the exposed inner layer and dissolve the target polymers there [19]. By selecting appropriate solvents and temperatures, the process is consecutively, and individually, applied to the different thermoplastic polymers present in mixed packaging plastic waste. The results are then used to calculate mass balances and to determine the resource potential of the different polymer types in each individual packaging waste fraction, even if they are not easily accessible due to the presence of other substances in mixed and multi-material packaging.

In parallel, DSC, microtome cuts, optical microscopy and FT-IR are used to determine the layer composition of selected packaging items per category and to validate the selective dissolution approach. This combined approach will provide new insights on the real recycling feedstock currently produced in the three European countries France, Germany and Belgium. It will allow to improve the existing sorting and recycling processes and cascades accordingly.

## 2. Materials and Methods

### 2.1. Categorisation of Waste Streams for Sample Description

Five bales of flexible plastic packaging were collected from three sorting sites in Europe, amounting, overall, to 2670 kg of plastic waste. The sorting sites in Europe were selected in countries (France, Belgium, Germany) with implemented extended collection and sorting of flexible packaging including screening, wind sifting, ballistic separation and NIR sorting. The plastic film fractions collected and sorted in France, Belgium and Germany (GER-310) were analysed, as well as two additional German sorting fractions from the sorting residue containing flexible packaging, flexible Mixed Polyolefin Items (GER-323-2) and New Mixed Plastics (GER-352). The German sorting fractions were based on the specifications of Der Grüne Punkt Holding GmbH & Co. KG, Köln, Germany [20,21,22].

**Figure 1:**
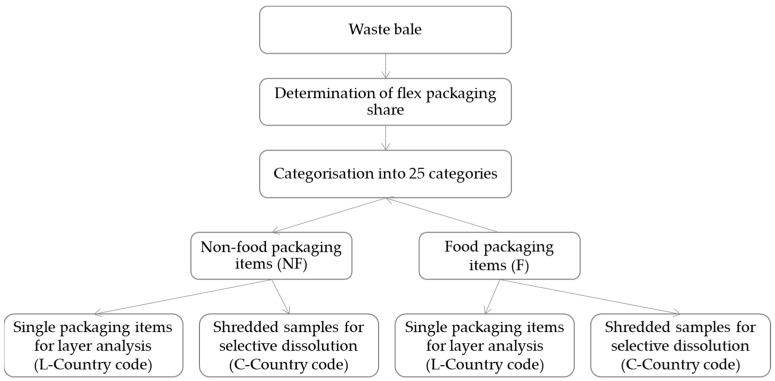
Scheme describing the sampling process in this paper.

The sorting fractions were characterised to determine the *flexible packaging share*: the choice of sample types and quantities as well as the management of the sampling plan were conducted based on the standard EN14899 [23] and two technical reports [24,25]. Masses between 60 kg and 200 kg were sampled according to the distribution of the waste in bales and in sorting refusals. The extraction of the packaging waste from compacted bales was performed manually after opening the bales. Subsequently, this flexible packaging was separated for detailed analysis from the remaining waste (non-flexible waste). This characterization into categories was performed according to the standards NF X30-408 (2020) [26] and NF X30-472 (2014) [27] and to the methodology MODECOM^TM^ [28]. For *polymer composition* analysis, each fraction of flexibles was separated into food (F) and non-food (NF) packaging per country. The specific packaging compositions and their *single-polymer layers* were analysed for the previously categorised packaging items after manual sampling according to the packaged good type (e.g., vegetables, fish, meat, ready-to-eat meal) and packaging film type (e.g., transparent, white, with aluminium/metallised). A minimum of one of each packaging item per good and packaging film type was chosen for characterisation to cover a broad range of packaging types. This sample selection was repeated for each category of every country to obtain a broad overview of the packaging items collected. In total, 294 items were chosen and analysed subsequently (95 items from France, 47 items from Belgium, 90 items from GER-310, 62 items from GER-323-2). While the French waste could be clustered into 25 categories, only 15 of these categories were found for the Belgian waste. An average of 3–4 samples per category were analysed for their layer composition. An overview about the sampling process is given in Figure 1.

Table 1 presents an overview of the waste bales from different sorting fractions that were characterised in detail, as well as the amount of material that was investigated and the share of flexible packaging material in each original waste bale. Furthermore, the amount and number of samples, which were further analysed by selective dissolution (C) and layer analysis (L) per country, as well as the respective sample identifiers (label), are shown.

### 2.2. Selective Dissolution for the Quantification of Polymer Shares in Waste Streams

In this study, the input waste was characterised by the content of typical polymers for packaging applications, i.e., PE, PP, polystyrene (PS), polyvinyl chloride (PVC), polyamide (PA) and polyethylene therephtalate (PET), using 6 specific dissolution conditions (solvent + temperature) [6,7]. To ensure a better homogeneity of the samples, 5 kg of each waste fraction, previously separated into food and non-food packaging, were shredded to a particle size of less than 1 cm side length in a Dynamic 25.38 cutting mill (Wanner Technik GmbH, Wertheim am Main, Germany), and 200 g of each waste stream was dissolved in duplicate for characterisation. For this purpose, the respective solvent was heated to the polymer-specific dissolving temperature for the recycling process in a 5 L beaker on a heating plate MR HEI-TEC Ø145 (Heidolph Instruments GmbH & Co. KG, Schwabach, Germany). The amount of solvent was selected in the respective dissolving step to obtain a solid/liquid ratio of 10%. The solution was mixed at constant temperature under constant stirring with an IKA^®^ EUROSTAR 20 digital stirrer (IKA-Werke GmbH & CO. KG, Staufen im Breisgau, Germany) for 30 min until the polymer was completely dissolved. It was then filtered with a cotton cloth, so that undissolved components were removed. The residue was rinsed with further solvent to remove soluble adhering residues. Subsequently, the solid content was determined by means of an MA35 moisture analyser (Sartorius AG, Göttingen, Germany) at 160 °C for 70 min, and the content of each target polymer in the solution was calculated via the mass of the filtered polymer solution. Based on these results, the mass of the respective selectively dissolved polymer was obtained. Afterwards, the residue was used as input for the next extraction step according to the same principle of selective dissolution. After the last dissolving step, the residue was dried in a VO400 vacuum oven (Memmert GmbH & Co. KG, Schwabach, Germany) and then weighed. The polymer fractions separated by selective dissolution were identified by Fourier transform infrared spectroscopy (FT-IR) and differential scanning calorimetry (DSC). If the analysis did not provide a clear identification, the mass fraction was attributed to the residue. The mass fractions $w_{i}$ of the individual polymers in the sample were determined via the measured masses $m_{i}$ and the sum of the masses of all polymers and the residue $m_{tot}$

(1) $$w_{i}=\frac{m_{i}}{m_{tot}}$$

### 2.3. Analytical Methods

The following analytical methods were applied to single flexible packaging items manually picked from the French (L-FRA) plastic film fraction, the Belgian plastic film fraction (L-BEL), the German plastic film fraction (L-GER-310) and the German mixed polyolefin item fraction (L-GER-323-2).

#### 2.3.1. Differential Scanning Calorimetry

To determine the polymer (composition) of a single packaging item or a dissolved sample, DSC analysis was performed with equipment from Mettler-Toledo GmbH (Gießen, Germany) according to ISO 11357 [29]. DSC measures the change in heat flow between a sample and an empty reference pan as a function of temperature. The measurement takes place according to a controlled program, increasing the temperature twice from 20 °C to 300 °C and decreasing it once, constantly at 10 K/min. The second heating run is used for the analysis. The phase transition of (semi-)crystalline polymers is measured and plotted as a function of temperature, whereas melting processes and crystallisation processes result in endothermic and exothermic peaks, respectively, which are characteristic of the analysed material. The maxima of the peaks are considered as the melting temperature *T_m_* and the crystallisation temperature *T_c_*, and the integral between reference pan and sample is the energy of the process consumed or released, respectively. A mix of different polymers shows superimposed melting curves. Depending on the superimposition, the polymer composition can be deducted if the peak temperatures can be distinguished. The curves of the melting temperature (range) and the melt enthalpy of the polymers hence allow for a qualitative, but not quantitative, identification of the polymers present in a sample.

#### 2.3.2. Fourier Transform Infrared Spectroscopy

To identify the polymers in a sample, a Fourier transform infrared spectroscope (FT-IR, Spectrum One, PerkinElmer LAS Germany GmbH, Rodgau, Germany) was used. For the film samples of the waste streams, the surfaces of the films were characterised on small samples of ca. 5 mm × 5 mm of packaging film in the instrument using Golden Gate ATR (attenuated total reflectance). For the dissolved sample identification, a sample of a similar size was cut and used. The polymer types were determined by comparing the measured FT-IR spectra with entries in a spectral library.

If both surfaces of one film sample showed the same material, in line with the enthalpy curves of DSC, the material was considered consisting of one polymer type, i.e., as a mono-material. The thickness of the film was then measured manually. If FT-IR and DSC showed more than one material (multi-material), a microtome cut was performed. DSC, FT-IR and microtome cut were used in combination to determine the main polymers and components in the structure.

#### 2.3.3. Optical Microscopy with Polarised Light on Microtome Cuts

For the identification of the layer structures and polymer layer thicknesses in the film samples, cross sections of the films made by microtome cuts were examined under the optical microscope Leitz Diaplan (Wetzlar, Germany). To obtain a 20 µm thin slice of the packaging film, a piece of the film was cut on a microtome cut device from Leica Instruments GmbH (Type: Jung Autocut 2055, Wetzlar, Germany) and placed on a carrier plate. With the optical microscope, a magnification of 200×–500× was possible, and with the integrated polarised light, the different materials in the film appeared in different colours, which made the layer structure clearly visible. With the corresponding software Leica Application Suite (LAS), version 4.12.0 (Build 86), the layer thicknesses were measured. To determine the polymer–layer shares, µm%, the total thickness of one polymer type, ${µm}_{i}$, was divided by the total thickness of all measured packaging items, ${µm}_{tot}$. Formula (2) is the calculation basis for the composition analysis of multi-layered films

(2) $$µm\%=\frac{{µm}_{i}}{{µm}_{tot}}\times 100$$

Additional identification of, e.g., EVOH-based barrier layers was possible by applying iodine on the microtome cuts. Iodine reacts with EVOH and makes it visible as a black layer under an optical microscope. 

## 3. Results

### 3.1. Categories Identified within the Waste Streams

**Figure 2:**
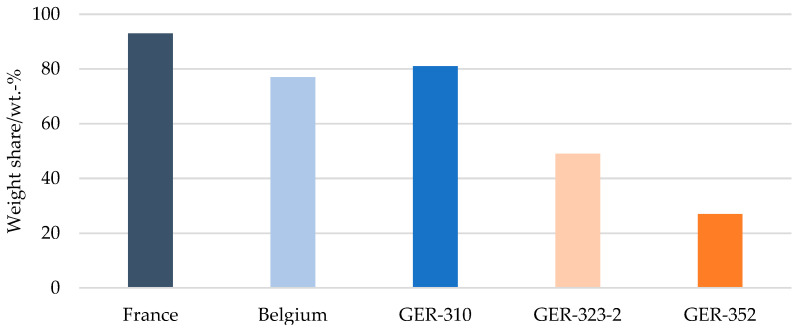
Flexible packaging share per country.

Manual sampling and sorting showed that the French plastic film waste fraction contained 93 wt.-% of flexible packaging, the Belgian plastic film fraction contained 77 wt.-% of flexible packaging, and the German PE film waste fraction GER-310 81 wt.-%. The German fraction GER-323-2, collecting flexible mixed polyolefin items, showed a flexible packaging share of 49 wt.-%, whereas the new mixed plastics fraction GER-352 comprised only 27 wt.-% of flexible packaging (ref. Figure 2).

The manually sorted flexible packaging items were further classified in food/non-food packaging. As a next step, they were clustered according to product type, obtaining 12 sub-categories identified in the flexible food packaging waste and 13 sub-categories in the flexible non-food packaging waste. The full list of sub-categories can be found in Table A1 in Appendix A.

**Figure 3:**
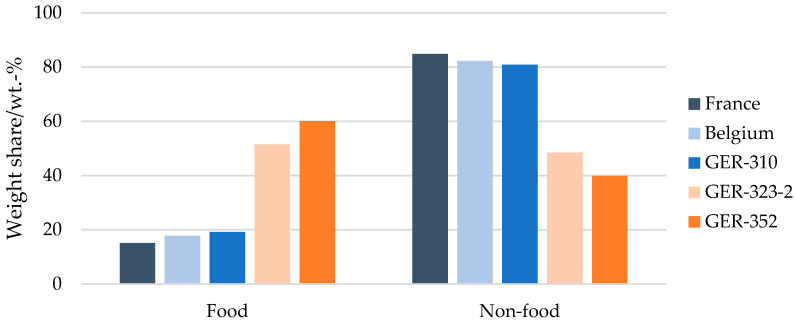
Comparison of the flexible packaging composition (food/non-food) of the bales from the 3 EU sorting sites.

Figure 3 shows the characterisation results of the flexible packaging in the five sorting fractions, split between food and non-food packaging. The Belgian, French and GER-310 bales contained similar wt.-% of food packaging, between 15 wt.-% and 18 wt.-%. The major type of flexible packaging constituting these bales was non-food packaging, amounting to more than 80 wt.-%. The contrary was observed for the other two German fractions, GER-323-2 and GER-352. The share of identified food packaging items was 50 wt.-% and 60 wt.-%, respectively.

A closer look was taken at the food and non-food fractions. The flexible food packaging waste included the same main sub-categories (frozen food (3–4 wt.-%), fresh products (2–3 wt.-%), bakery products (~2 wt.-%)) in all three plastic film fractions from France, Belgium and Germany, with a similar order of magnitude in weight-%. Exceptions were the sub-categories of ready meal and sweet and snack packaging, which showed double amounts in the GER-310 and in the Belgian bales compared to the French ones. The three main categories found in GER-323-2 and GER-352 were sweets and snacks, fresh products and convenience food (8–11 wt.-%).

For flexible non-food packaging, collection bags represented a high share of the plastic film fractions in all countries, due to the collection scheme established (15–22 wt.-%). Categorisation also showed the presence of 15 wt.-% of plastic tarps in the Belgian bales, due to the possibility to dispose of such waste in blue collection bags in Belgium. The share of secondary industrial packaging, consisting in transparent films from stretch film applications, was also relatively high in all three countries, but especially in the German fractions, representing the largest share in the respective fractions, corresponding to 14–38 wt.-%. The higher mass percentage of industrial films in the GER-310 compared to the French and the Belgian bales can be attributed to the bigger and thicker films found in the German bales. Their source could be the collection at companies, where large films are disposed of.

A detailed overview of the shares by product category is shown in Table A1 in Appendix A.

### 3.2. Quantification of the Polymer Shares in Flexible Packaging

**Figure 4:**
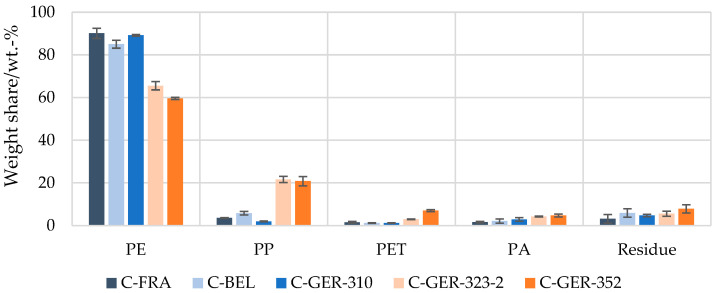
Mean value of polymer composition of flexible packaging waste in sorting fractions from France, Belgium and Germany determined by selective dissolution.

Investigation of the polymer shares in the European waste streams by this method revealed that the PE content in the flexible packaging waste fractions from France, Germany (C-GER-310) and Belgium were in the same range of 85–90 wt.-%, with the lowest PE share in the Belgian waste fraction, corresponding to 85 wt.-% (Figure 4). The DSC analysis showed that all fractions contained a mixture of PE of different grades (LDPE, LLDPE, HDPE). Shares of 1–6 wt.-% of PP, PET and PA were measured in all flexible packaging waste fractions. The purity of the selectively dissolved polymer fractions was identified and verified successfully with the help of FT-IR and DSC measurements. Using a selective solvent for PS and PVC, dissolved fractions of 0–3 wt.-% were measured. However, the analysis could not clearly identify the dissolved polymers as PS or PVC. The FT-IR pattern showed a clear OH adsorption band, suggesting that a mixture of other rather non-polar barrier polymers such as EVOH were dissolved. The determined mass fractions were therefore attributed to the residue. In addition to the polymer shares that could not be clearly identified, a residue of 1–4 wt.-% also remained undissolved after all the dissolution steps carried out. This was largely composed of paper and aluminium but also contained smaller amounts of adhesives, glass or wood, according to visual inspection.

In the flexible packaging waste originating from other German sorting fractions (C-GER-323-2 and C-GER-352), a distinctly lower PE share of 60–66 wt.-% was measured. At the same time, a distinctly higher PP share of 21–22 wt.-% was found. PET and PA were determined at 3–7 wt.-%, and their shares were thus in the range of those of the other sorting fractions considered.

**Figure 5:**
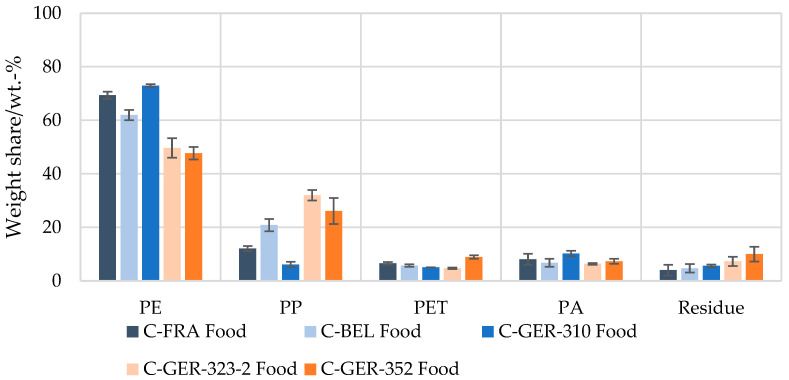
Mean value of polymer composition of flexible food packaging waste in sorting fractions from France, Belgium and Germany determined by selective dissolution.

**Figure 6:**
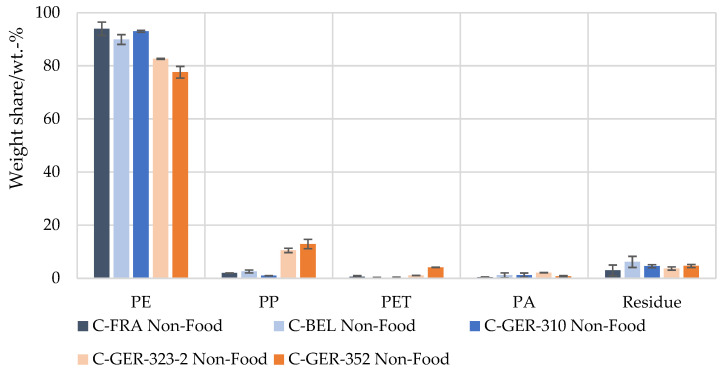
Mean value of polymer composition of flexible non-food packaging waste in sorting fractions from France, Belgium, and Germany determined by selective dissolution.

Clear differences were seen in the composition of food and non-food packaging (Figure 5 and Figure 6). For non-food packaging, very high PE contents of up to 94 wt.-% were measured, with lower values for the Belgian fraction and the German fractions C-GER-323-2 and C-GER-352, as described above. Food packaging was characterised by a distinctly higher variance in polymer composition. PE proportions of 48–73 wt.-% were measured in these fractions, while the remainder was composed of up to 32 wt.-% of PP and up to 10 wt.-% of PET and PA.

### 3.3. Identification and Quantification of Polymers in Flexible Packaging

**Figure 7:**
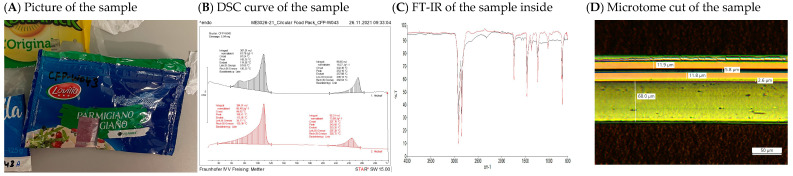
Exemplary collection of analytical results of a printed cheese film (Parmigiano). (**A**) picture of the exemplary sample. (**B**) DSC curve, black line shows the first heating run, read line show the second heating run. (**C**) FT-IR spectrum with red line representing the sample inside result, and black line showing the data bank result for PE. (**D**) Microscopic image of the sample’s microtome cut.

The polymer layer composition of the selected items was determined by combining the analytical results of DSC, FT-IR and optical microscopy (with polarised light). Figure 7 shows an example of a DSC and microtome cut analysis combination: the DSC graph in B shows the curves of the first (black) and the second (red) heating run. The first and larger peak visible indicates the presence of LDPE (T_m_ of 108 °C), the second smaller peak the presence of PET (T_m_ of 244 °C). Moreover, it could be seen by inspecting the samples that there seemed to be aluminium or metallisation (metallic shine inside the samples). The FT-IR spectrum indicated PET on the outside and PE on the inside of the sample, the latter is shown in Figure 7C, where the spectrum obtained for the presented sample is indicated by the red line in comparison to the black spectrum corresponding to the PE spectrum according to the data bank used. By soaking the film piece in ethyl acetate, delamination can be provoked to reach the inner film layers and confirm the material determination via FT-IR. This led to the conclusion that the sample shown in Figure 7A was a multi-layer multi-material film. The layer thicknesses were measured on microscopic images of the microtome cuts, as shown in Figure 7D; the polarised light used made the materials appear in different colours due to their different refractive indices. It could, hence, be concluded that the sample consisted of printed PET on the outside (11.9 µm), laminated to metallised PET in the middle (11.8 µm) and laminated against transparent LDPE as a sealant layer on the inside (68.0 µm). The combination of these investigations allowed for the determination of whether the sample could be clustered as a mono- or a multi-layer film and as a mono- or a multi-material. This way, the composition of every single layer in the investigated packaging items was determined per fraction, as previously indicated in Table 1.

**Figure 8:**
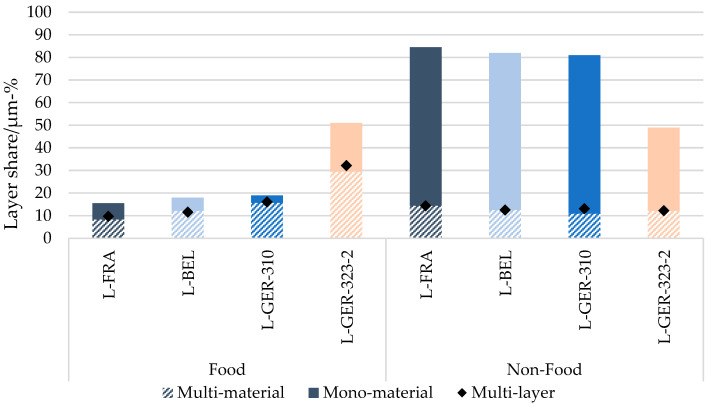
Clustering of the analysed samples into multi-material, mono-material and multi-layer, in percentages of the total number of samples per food and non-food section.

Figure 8 shows the allocation to mono- or multi-material and multi-layer of the flexible packaging waste per country, divided into food (F) and non-food (NF) packaging, according to the initially determined overall share found in the fractions (ref. to Figure 3). A multi-material is a film comprising several polymers, and a mono-material is a film consisting of one single polymer. Multi-layer packaging films consist of several layers combined by adhesive layers visible in microtome cuts. It is probable that the number of multi-material packaging items and multi-layer packaging items differs, because a multi-layer film can consist of two films of the same polymer type making it a mono-material but a multi-layer, and a multi-material film can consist of two different polymer types coextruded in one film.

The food packaging samples L-FRA, L-BEL and L-GER-323-2 showed similar multi-material shares of 50–70%; the food packaging sample L-GER-310 had a higher multi-material percentage of 83%. The share of multi-layer films was slightly higher, within the 63–84% range. The non-food packaging, on the other hand, showed a lower multi-material content of 15–25%.

The share of multi-material and multi-layer packaging items was in all countries higher in food packages than in non-food packages relative to the total shares. There were only slight differences between the number of multi-materials and the number of multi-layers. The figure shows that in the food section of the currently sorted waste streams, the share of multi-materials and multi-layers was dominant.

**Figure 9:**
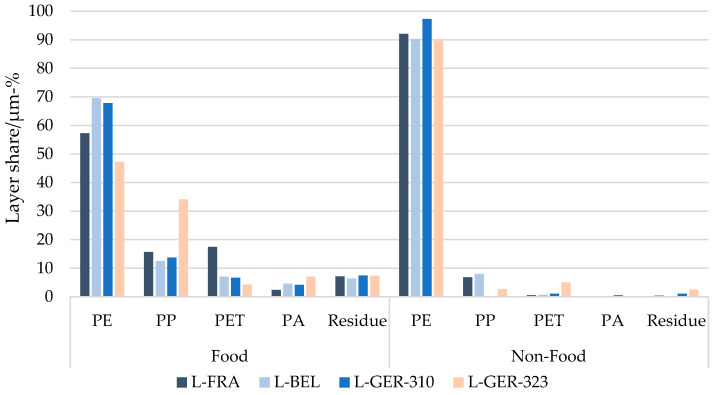
Measured layer thickness composition of the 294 analysed flexible packaging items from France, Belgium and Germany in layer percentage [µm-%].

The detailed analysis of the packaging items selected from the sorting fractions is depicted in Figure 9, which shows the polymer share according to the layer thicknesses measured in the 294 items and in combination with the polymers found by selective dissolution (Figure 3 and Figure 4). It allows for a more comprehensive look at the specific items.

PE was the most abundant polymer in the food packaging items, with a share of up to 70%, followed by PP (up to 34%), PE and PA. The same polymers were found in the layers of the non-food packaging items analysed, with PE being the dominant component (up to 97%), and PP, PET and PA accounting in total for less than 10 µm-%.

The analysis of the single layers in the packaging items allowed for a specification of what is called “residues” in Figure 4 and Figure 9. Aluminium, EVOH, adhesive and lacquers, tie layers and papers built up to a share of residues up to 7 µm-% in the total food packaging analysis performed, but below 3 µm-% in the non-food items.

**Figure 10:**
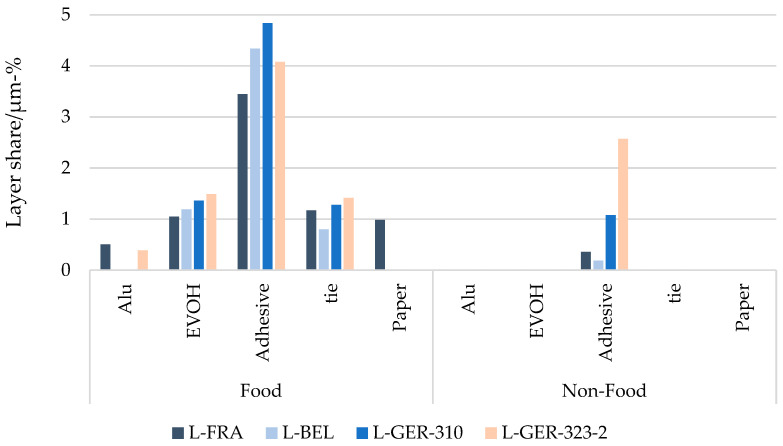
Residue amount at a “higher resolution” to identify the materials contained in the food and non-food packaging items of the different fractions.

Figure 10 shows the residue composition at higher resolution. The residues identified in non-food packaging were merely clustered as adhesives, while the food items contained a variety of different residue materials: EVOH as a barrier polymer and tie layers needed, e.g., to produce PE/EVOH/PE coextruded films, as well as aluminium and paper were found. Thin inorganic coatings could not be determined with the methods applied because the layer thickness was on the nanometre scale; so, no conclusion can be drawn for these barrier types. The high share of adhesives compared to those of the other materials in the food items was in line with the relatively high share of multi-layer packaging found in these packages. Additionally, most of the food packaging found in the German fractions was printed, amounting to more than 50%. The measured shares of paper in the residue composition were therefore comparatively low and only present in the French food fraction. The sampling for the selective dissolution described in Section 3.2., however, represented a more homogeneous mixture of waste, which is why paper was identified there in the residues of all fractions but could not be quantified due to the impossibility of separating the residues with this method.

## 4. Discussion

### 4.1. Flexible Packaging in European Waste Streams for Polyolefins and Lightweight Packaging

The results of the analyses showed that the PE film fractions sorted in the three European countries differed significantly in polymer content and flexible packaging shares, despite the common NIR sorting according to PE. All fractions contained more than 10 wt.-% of foreign (non-PE) materials, while flexible packaging amounted to 76–92 wt.-% of the PE film fractions. The determined 81 wt.-% of flexible packaging in GER-310 lies slightly below the 88 wt.-% reported 2011 in [30] and considerably below the specification of 92 wt.-% from the DSD [21]. The mixed polyolefin item fraction GER-323-2 should contain flexible packaging items like films and bags, but also rigid items made of polyolefins, like trays, lids, caps or labels, resulting in a maximum of 15 wt.-% of impurities [22]. The detailed analysis focused on the flexible items in this stream making up around 48 wt.-% of the stream, the selective dissolution method determined 88 wt.-% of polyolefins (59–65 wt.-% of PE and ~21 wt.-% of PP) in C-GER-323-2 and a PET share below 5 wt.-%. These results lie within the specifications reported by Der Grüne Punkt Holding GmbH & Co. KG for this stream. However, it only offers insights on the flexible packaging within this fraction, while the composition of the rigid packaging sorted into GER-323-2 remains unknown and could be further investigated with a similar method as that described in this paper.

The waste investigated was collected at similar times of the year, which means that seasonal variation was not considered in the analysis. However, the results provide a good overview of the variation between countries, which, even with well-established collection and sorting systems, shows potential for improvement and points to the need of European harmonisation.

### 4.2. Selective Dissolution to Determine the Potential Recycling Pathways for Waste Streams

The selective dissolution method for the compositional analysis of the flexible packaging waste resulted in up to 7 wt.-% of residues comprising indissoluble materials. The analysis of the layers identified those as aluminium, EVOH, adhesives, lacquers or inks and tie layers, as well as paper. The residue share was especially high in the food packaging category. Aluminium or a metallisation is often used as a gas and light barrier, while EVOH is a barrier polymer used to obtain a good oxygen barrier in food packaging. Adhesives are required to combine different polymers in multi-layer multi-material films often used for food packaging, while lacquers and inks are needed for decoration on the packaging, which is also usually more common for food packaging than for non-food packaging, with the exception of cosmetics or home and personal care products. The residues identified in non-food packaging, however, were merely adhesives, as found in cosmetic packs. It should be noted that it was not always possible to differentiate between a lacquer or ink and an adhesive, both based on polyurethane, which is why they were evaluated in aggregate.

When it comes to the PP share, it was determined that in the PE flexible fractions, the PP share remained below 10 wt.-% but increased up to 20 wt.-% when looking at the mixed flexible polyolefin fractions. Luijsterburg et al. investigated the composition of recyclates from different sorting fractions of German and Dutch sorting plants with FT-IR and DSC in 2013 [31]. They found that 7–17% of PP was contained in the PE film fractions. However, the results of the present study showed a higher percentage, and it can clearly be stated that the high PP share derived from the food packaging. As PP was the second dominant polymer found in this fraction, it follows that PP films are mainly used for smaller packaging applications like pouches or flow packs, compared to PE. The non-food items contained significantly less foreign polymers.

With the presented method of selective dissolution, larger and well homogenised samples of post-consumer flexible packaging waste fractions were analysed precisely for their polymer composition for the first time, considering the polymers both in mono-material and in multi-material structures. It could be shown that the method for the composition analysis of waste can provide insights into the sorting quality of waste fractions. To make use of such a potential polymer resource, solutions are required to overcome the hurdle of material mixtures and multi-material structures, which currently cause such fractions to only qualify for non-packaging applications or energy recovery. Pre-treatment and recycling technologies allowing for the separation of polymers in multi-layer structures are evolving, like solvent-based dissolution, delamination techniques and, increasingly, chemical recycling. [32,33] However, for thermolytic chemical recycling like pyrolysis, a minimum requirement of 85% of PE and PP content in the feedstock has been reported [34], which is a high purity, considering the existing material mixtures reported in this paper, e.g., in Figure 5.

Based on the presented results, it can be derived that all five waste streams contained a reasonably high share of PE and PP and may be an interesting feedstock for recycling. However, mechanical approaches need to involve advanced technologies like delamination. Solvent-based technologies may separate purified polymers from mixtures and multi-layer structures, as exemplified in these laboratory experiments. In both cases, the respective expected recycling yields can be derived from the presented polymer content.

### 4.3. Multi-Layer Packaging Content in European PE Film Fractions up to 29 µm-%

The investigation of the layer structures revealed a multi-layer share of 24–29 µm-% in the PE film fractions sorted in the three countries (L-FRA, L-BEL and L-GER-310) and of 44.5 µm-% in the L-GER-323-2 flexible, mixed polyolefin item fraction. With the simplified assumption of polymer densities around 1.0 g/cm^3^, a link to the wt.-% results from other studies is viable. Schmidt et al. determined a multi-layer share of 43.2 wt.-% in 1190 randomly selected items from lightweight packaging collected in German households before any sorting [35]. Considering the sample selection of Schmidt et al. and assuming that most of these packages would be found in GER-323-2 after a sorting step due to size or material identification, these results seem comparable to ours. In the CEFLEX study on European waste composition, 22–26% of multi-material combinations were found in German and French flexibles from the recyclable stream [18]. The present study specified that most multi-material packaging items corresponded to food packaging with up to 83 µm-%.

The large share of multi-materials found in the analysis can be traced back to the requirements of protection from oxygen, light or moisture of most food products. The smaller share of multi-material and multi-layer packaging in the non-food products derived from sensitive products requiring chemical barriers like disinfecting wipes; however, the larger share was for mono-materials.

PP, PET or PA were the main polymers found and are considered incompatible with PE, e.g., due to different melting temperatures. Non-polymeric materials such as aluminium or materials with a high quantity of paper are also used in multi-layer packaging. This leads to a complex material mix in recycling and subsequently to poorer quality [14]. The application fields for such material mixtures are limited, and large amounts of recyclates are used in other products than packaging and can therefore not be circulated in the same product field or at all [30].

To reduce incompatible material mixtures, designing packaging for recycling has been suggested and performed for several years. Guidelines have been established and made available on the percentage of packaging that should be made of the same material; guidance for additives, barrier materials and colours exists, and certifications for recyclability [36,37,38,39] were established to incentivise brand owners and material producers to make changes in packaging design. One design key is to mostly use a mono-material as packaging material, with a foreign material share below 10 wt.-% [37]. According to a recent study on flexible food packaging in Europe, the consumption of PE-, PP- and OPP-based films is expected to grow in the coming years, while the use of aluminium-based films as well as PA-based films is expected to be reduced [40]. Subsequently, the share of multi-material packaging in general could be reduced.

### 4.4. Potential of the Distinguished Sorting and Separation of Food and Non-Food Packaging

Besides the interesting differences between food and non-food packaging, the analysis in this study further confirmed that the multi-material packaging content is especially high in food packaging, with 118 out of 179 items (65%) presenting multi-material packaging, compared to 20 out of 115 non-food packaging items (17%). These food products usually require a barrier against oxygen, moisture and light to protect the product and avoid any spoilage. The resulting high diversity of materials in food packaging waste, which are inseparable in the existing infrastructure mainly based on mechanical recycling, hampers high-quality recycling and leads to a low recyclate quality. In this context, this study provides interesting insights in potential treatment options for food packaging (waste) to make it a more valuable resource:

- A redesign of the current multi-materials could reduce material variety.

Designing food packaging with a lower variety of materials would help to improve the recyclate quality. Thin barrier coatings can achieve similar barrier performance for the protection of packed goods as the current barrier film structures like PET-PE laminates. Moreover, a reduction in the printing colours and the choice of the lamination adhesive can influence the recyclability of packaging films by reducing the use of PET, PA and adhesive, which could remove the main impurities from the current food packaging and improve the sorting outcome.

- Additional sorting will increase the recycling feedstock of plastic packaging.

The analysis of the three German sorting fractions with different composition specifications showed the potential of extensive lightweight packaging sorting. It was also shown that an extra sorting step can lead to additional fractions with a PE share of >60 wt.-% and a share of polyolefins (PE and PP) of >80 wt.-%, as shown for Germany GER-323-2. The number of lightweight packaging sorting facilities has increased in the past two years in Germany, where 69 of the existing 366 plastic packaging waste sorting facilities also sort lightweight packaging, with only one of them sorting flexible waste according to colour (natural and coloured LDPE, PP and mixed polyolefins) [41,42]. In countries without dedicated sorting fractions like those of mixed polyolefins, such multi-layer food or cosmetic packages are incinerated as refusals and lost for a circular economy approach. Plastics Europe states that the recycling rates can be increased by a factor of 13 when plastic waste is collected via separate waste collection systems where consumers collect their waste according to its usage (electronics, packaging waste, paper, etc.) [7]. The positive effect of additional sorting was also investigated, e.g., by Gadaleta et al. and Kusch et al. They calculated and modelled material streams with the results of improved sorting efficiency, a higher recyclates yield and an improved environmental impact compared to those obtained with the current sorting steps [43,44].

- Additional improvement in the food and non-food packaging recycling feedstock.

Furthermore, separating food from non-food packaging items specifically could produce two types of improved feedstocks: the quality of the non-food packaging stream would increase due to the lower content of non-PE and multi-materials (ref. Figure 8); a pure food packaging stream would bear the potential of being used again for food packaging. The reuse of recycled material is regulated and limited by EC 2022/1616. It is stated that the origin of recycled material intended to be used in food contact applications must be a food contact application. This constraint is based on the strict positive list of substances allowed in such material [12].

Concepts like integrating photoluminescent markers or digital codes in the décor of packaging are being developed to improve the sorting of packaging waste. In research, different options of markers excitable via specific light or X-ray sources have been developed. They may potentially change the sorting efficiency of waste, e.g., of packaging, by enabling the definition of more precise sorting categories than those achievable by spectroscopic material identification only [45].

A major challenge in the recycling of flexible food packaging is the presence of contaminations, known and unknown, leading to malodour and potentially harmful substances. Roosen et al. showed that odorous pollution is significantly lower in non-food packaging than in food packaging [46], which in turn also contains less inks and a lower variety of polymers. A simpler design of the packaging for improved circularity could hence be of large value to the recyclate quality.

It was shown that flexible packaging in the film bales contained a high PE share and had limited printing, consisting mainly of non-food packaging. These PE film fractions containing up to 90 wt.-% of PE are potentially a good feedstock for mechanical recycling without major discoloration due to their limited printing. By explicitly separating the non-food packaging from the food packaging, the PE share in non-food packaging could be increased to 94 wt.-%, making it an even more valuable feedstock.

## 5. Conclusions

In the frame of this work, the composition of flexible packaging waste was shown to vary between the considered countries, despite similar sorting facilities. The output of three sorting sites in France, Belgium and Germany was selected, representing the existing PE sorting processes used in many European countries. The collected packaging waste sampled for this study corresponds to the currently existing waste feedstock collected for recycling in countries with implemented extended lightweight packaging collection systems put in place by extended producer responsibility (EPR) systems. The selected fractions presented in this study showed the potential of specifically collecting lightweight packaging via EPR systems and can serve as an example for other European countries to homogenise flexible packaging waste streams throughout Europe. This study also shows the necessity of aligning and harmonizing regulations and infrastructure throughout Europe to increase the availability of recycling facilities and high-quality recyclates.

As the current legislation stipulates that recycled packaging that comes from food applications should only be used in “closed-loop” recycling for novel food packaging, there is a crucial demand of suitable solutions for sorting food from non-food packaging items. Moreover, novel recycling technologies are required in order to achieve high-quality recyclates, addressing the variety of the materials used by a better separation of multi-layers and the purification of the included contaminants.

The studied waste examples show that by sorting mixed polyolefins (see GER-323-2), flexible food packaging items are enriched in this specific sorting fraction up to 50–60 wt.-%, suggesting that it could be used as feedstock for the additional elimination of non-food items. Thus, a “food-only” fraction might be produced, which is key to any downstream (and still visionary) processing to obtain highly purified polymers suitable for food packaging, even if the packaging in such a food fraction would still consist of a large variety of material types besides PE (PP: 8–32 wt.-%, PA: 5–10 wt.-%, PET: 5–10 wt.-%). However, technologies tailored to this input stream like delamination and/or solvent-based recycling could be further developed, upscaled and implemented. With a better specified input waste stream, a more specified output recyclate of higher quality could be achieved and potentially allow for a circular use in flexible food packaging.

The data presented offer insights on the detailed composition of a broad mix of flexible packaging items from different European collection and sorting streams. Based on these data, novel recycling and pre- or post-treatment technologies can be adjusted to and combined in cascades in the existing streams, to keep more resources inside the system. The data also present changes in feedstock for recycling when reducing the refusal fractions by additional sorting. By re-designing the packaging material, the streams intended to be incinerated can also be reduced. Packaging made of one material only would consequently contribute to improving the recyclate quality and enabling a more circular economy.

This study also makes it clear that valuable resources mainly trapped in food packaging are sorted into a refusal fraction in France and Belgium due to their size. The German examples investigated show that if items smaller than A4, which were separated from the French and Belgian flexible waste and GER-310 fractions, are also collected and sorted (GER-323-2), the food packaging streams can be enriched and made available for further processing steps.

The methods used contribute to a better understanding of the European waste streams, which is necessary as a basis for the targeted development of recovery and recycling concepts for high-quality recyclates and a circular economy.

## Figures and Tables

**Table 1:** Overview of sampled waste masses and their distribution into food and non-food (NF) packaging, packaging items thereof and sample naming.

Materials Bales References	Plastic Films France (France)	Plastic Films Belgium (Belgium)	Plastic Films Germany (GER-310)	Mixed Polyolefin Items Germany (GER-323-2)	New Mixed Plastics Germany (GER-352)	Total
Initial mass of the bales	560 kg	400 kg	550 kg	720 kg	440 kg	2670 kg
Sampled mass for investigation	277 kg	180 kg	64 kg	108 kg	190 kg	819 kg
Mass of flexible packaging	257 kg	138 kg	51.9 kg	52.6 kg	51.4 kg	550.9 kg
Mass% of flexible packaging	92.8%	76.7%	81.1%	48.7%	27.0%	-
Label for selective dissolution results	C-FRA	C-BEL	C-GER-310	C-GER-323-2	C-GER-352	-
Mass for analysis by selective dissolution	Non-food per waste stream: 0.4 kg					4 kg
	Food per waste stream: 0.4 kg					
Label for layer composition results	L-FRA	L-BEL	L-GER-310	L-GER-323-2	-	-
Packaging items for layer analysis	95 pcs.	47 pcs.	90 pcs.	62 pcs.	-	294 pcs.

## Data Availability

The original contributions presented in the study are included in the article, further inquiries can be directed to the corresponding author.

## Appendix A

**Table A1:** Mass shares of food and non-food packaging per country and respective sub-categories.

	France	Belgium	GER-310	GER-323-2	GER-352
Flexible Packaging Categories	Wt.-%	Wt.-%	Wt.-%	Wt.-%	Wt.-%
**Food Categories**	15.5	17.8	19.1	51.5	60.1
Frozen food	4.40	2.90	4.10	6.80	2.80
Bakery products	2.00	1.70	1.70	3.70	4.40
Meat and fish products	1.80	1.50	1.20	2.80	4.90
Fresh products (fruits, vegetables)	1.70	2.90	2.90	8.20	8.60
Chilled convenience food and ready meals	1.40	2.50	4.30	9.30	7.90
Pet food, pet supplies	1.20	1.70	1.30	1.60	5.90
Sweets, snack products	0.90	1.90	0.60	8.70	11.40
Nutriments, instant dry products	0.70	0.90	1.20	5.30	5.90
Dairy products	0.60	0.90	1.00	3.80	3.80
Other liquid and pasty food	0.20	0.60	0.80	0.70	1.00
Coffee, tea, cacao	0.10	0.20		0.60	3.50
**Non-Food categories**	84.5	82.2	80.9	48.5	39.9
Secondary packaging films (beverages)	23.00	18.10	4.90	4.40	1.50
Collection bags	20.40	22.40	15.40	20.50	8.60
Secondary packaging films (industry)	18.90	12.80	38.20	20.50	14.30
Carrier bags	6.40	3.20	4.00	6.30	7.00
Secondary packaging films (care)	6.80	2.50	6.20	7.80	5.50
Bags above 25 Litres	3.90	4.40	6.90		1.00
Stretch/bubble/foam films	3.60	2.50	3.30	2.10	
Secondary packaging films (newspaper)	1.70	0.20			
Detergents, cleaning agents	0.20	1.20			
Plastic tarp			2.00	0.70	
